# Prehispanic Use of Chili Peppers in Chiapas, Mexico

**DOI:** 10.1371/journal.pone.0079013

**Published:** 2013-11-13

**Authors:** Terry G. Powis, Emiliano Gallaga Murrieta, Richard Lesure, Roberto Lopez Bravo, Louis Grivetti, Heidi Kucera, Nilesh W. Gaikwad

**Affiliations:** 1 Department of Geography and Anthropology, Kennesaw State University, Kennesaw, Georgia, United States of America; 2 Department of Tourism Administration, Universidad Autonoma de Chiapas (UNACH), Tuxtla Gutierrez, Mexico; 3 Department of Anthropology, University of California Los Angeles, Los Angeles, California, United States of America; 4 Instituto Nacional de Antropología e Historia-Chiapas, Tuxtla Gutierrez, Mexico; 5 Department of Nutrition, University of California Davis, Davis, California, United States of America; 6 Departments of Nutrition and Environmental Toxicology, University of California Davis, Davis, California, United States of America; 7 Departments of Nutrition and Environmental Toxicology, University of California Davis, Davis, California, United States of America; 8 Department of Anthropology, University of Arizona, Tucson, Arizona, United States of America; New York State Museum, United States of America

## Abstract

The genus *Capsicum* is New World in origin and represents a complex of a wide variety of both wild and domesticated taxa. Peppers or fruits of *Capsicum* species rarely have been identified in the paleoethnobotanical record in either Meso- or South America. We report here confirmation of *Capsicum* sp. residues from pottery samples excavated at Chiapa de Corzo in southern Mexico dated from Middle to Late Preclassic periods (400 BCE to 300 CE). Residues from 13 different pottery types were collected and extracted using standard techniques. Presence of *Capsicum* was confirmed by ultra-performance liquid chromatography (UPLC)/MS-MS Analysis. Five pottery types exhibited chemical peaks for *Capsicum* when compared to the standard (dihydrocapsaicin). No peaks were observed in the remaining eight samples. Results of the chemical extractions provide conclusive evidence for *Capsicum* use at Chiapas de Corzo during a 700 year period (400 BCE–300 CE). Presence of *Capsicum* in different types of culinary-associated pottery raises questions how chili pepper could have been used during this early time period. As Pre-Columbian cacao products sometimes were flavored using *Capsicum*, the same pottery sample set was tested for evidence of cacao using a theobromine marker: these results were negative. As each vessel that tested positive for *Capsicum* had a culinary use we suggest here the possibility that chili residues from the Chiapas de Corzo pottery samples reflect either paste or beverage preparations for religious, festival, or every day culinary use. Alternatively, some vessels that tested positive merely could have been used to store peppers. Most interesting from an archaeological context was the presence of *Capsicum* residue obtained from a spouted jar, a pottery type previously thought only to be used for pouring liquids.

## Introduction

Upon reaching the New World, Christopher Columbus was one of the first Europeans to encounter the fruits of *Capsicum* species, calling them “peppers” because they had a spicy hot taste unlike anything else in Europe at the time. Shortly thereafter chili peppers were being added to dishes prepared in Spain and Portugal and their culinary use soon spread across Europe and into Asia. While these peppers likely had significance in the culinary culture of the New World at the time of Columbus’ voyage [1], the occurrence of cultivated chili peppers (*Capsicum* spp.) in earlier times, especially in Mesoamerica, is limited. Our knowledge of the use of chili peppers among Mesoamerican groups, such as the Maya, Olmec, and Zoque is limited at best. In these areas, *Capsicum* rarely has been identified in the paleoethnobotanical record compared to the presence of other foodstuffs like beans, maize, manioc, and squash. While archaeobotanists and archaeologists today have a much clearer picture of the range of foods used by ancient peoples of Mesoamerica in their daily subsistence and dietary practices, the information on the nature, extent, structure, and timing of chili pepper use or consumption in the archaeological record remains scant.

The genus *Capsicum* is New World in origin and contains a complex of 20–30 wild species and five domesticated taxa: *C. annuum*, *C. baccatum*, *C. chinense*, *C. frutescens*, and *C. pubescens* [2]. Of the five domesticated species of chili pepper, *C. baccatum* and *C. chinense* initially were domesticated in northern South America while it is probable that *C. annuum*, *C. frutescens*, and *C. pubescens* initially were domesticated in Mexico or northern Central America [2]–[4].

In South America, researchers have identified starch grains of *Capsicum* on artifacts, from milling stones and cooking vessels recovered from house floors in two early village sites at Loma Alta and Real Alto in southwestern Ecuador to ca. 6,000 years ago [3]. These microfossil remains represent some of the earliest dated chili peppers in the New World. Fruits from *C. baccatum* and *C. chinense* in early levels from two coastal Peruvian sites at Huaca Prieta and Punta Grande were dated to ca. 3800 years ago [4].

In Mesoamerica, Perry and Flannery [5] tentatively identified 8,000 year old stems, possibly from harvested from wild chilis at Guila Naquitz, a dry cave located in Oaxaca, Mexico. More convincing evidence, however, was found at Coxcatlan Cave in the Tehuacan Valley, dated to ca. 6,000 years ago [6]. The macrofossil data from the Tehuacan Valley site indicates that *C. annuum* was domesticated in Mexico at approximately the same time as the domestication of other *Capsicum* species in Ecuador.

Domesticated chili peppers also have been recovered from Guila Naquitz and the nearby site of Silvia’s Cave located in Oaxaca, Mexico. The excavated cave floors yielded a collection of 122 chili peppers that could be dated from CE 600-1521. These well-preserved specimens belonged to both *C. annuum* and *C. frutescens* and were found along with other domesticates including avocados, beans, maize, and squash. The remains may represent refuse discarded by work groups who camped in the caves for short periods of time while away from their village [5]. During excavations in the tunnel under the Pyramid of the Sun at Teotihuacan, researchers identified macrofossil remains of *Capsicum* sp. in construction fill dating to CE 150–250 [7].

Minnis and Whalen [8] reported first evidence for cultivated chili, identified as *C. annuum*, from a site near Casas Grandes/Paquime in northwestern Chihuahua dating to CE1200–1450. This charred specimen was excavated from a subfloor trash deposit in a room at Site 315 located approximately two kilometers from the Casas Grandes site.

In contrast to these finds, recovery of chili peppers from archaeological sites in the Maya area has been rare. Based on archaeological and linguistic evidence, Colunga-Garcia Marin and Zizumbo-Villarreal [9] have indicated that chili was cultivated by 1700 BC, if not earlier. Lentz [10] concluded that by at least 1200 BCE the ancient Maya had a maize-based system of food production that included beans, peppers, and squash. Archaeologically, the earliest example of chili in the Maya area comes from finds associated with Phase II (1000-400 BCE) deposits at Cuello in northern Belize. This evidence, however, is scant since only one seed of wild *Capsicum sp.* was recovered using flotation methods [11]–[12]. The Cuello site also revealed wood charcoal from domesticated chili that was recovered from a sealed chultun (subterranean storage feature) dated to the Late Preclassic (CE 100–200) period [13]. Another seed, identified as *Capsicum* sp., was found at the Late Preclassic site of Cerros in northern Belize [14]. Carbonized peduncles of *C. annuum* have been identified at Dos Pilas another Late Classic site in Guatemala [15]. The strongest chili-related archaeological evidence reported, however, comes from the site of Ceren in El Salvador where carbonized seeds, peduncles, and rinds of *C. annuum* were found in great abundance, especially in storage rooms and in a kitchen area where they were suspended from rafters in large clusters. These carbonized remains were well-preserved by the ash and lava from the Loma Caldera volcanic eruption in CE 540 [16].

Based on the data presented above, there are relatively few sites in Mesoamerica, Central America, and South America that contain remains of *Capsicum*. All of the specimens recovered to date are examples of microfossils or macrofossils. No chemical extractions have been performed on artifacts, whether pottery vessels or stone tools, to determine the consumption of chili peppers at sites in either area.

### Chiapa de Corzo Pottery Samples

**Figure 1:**
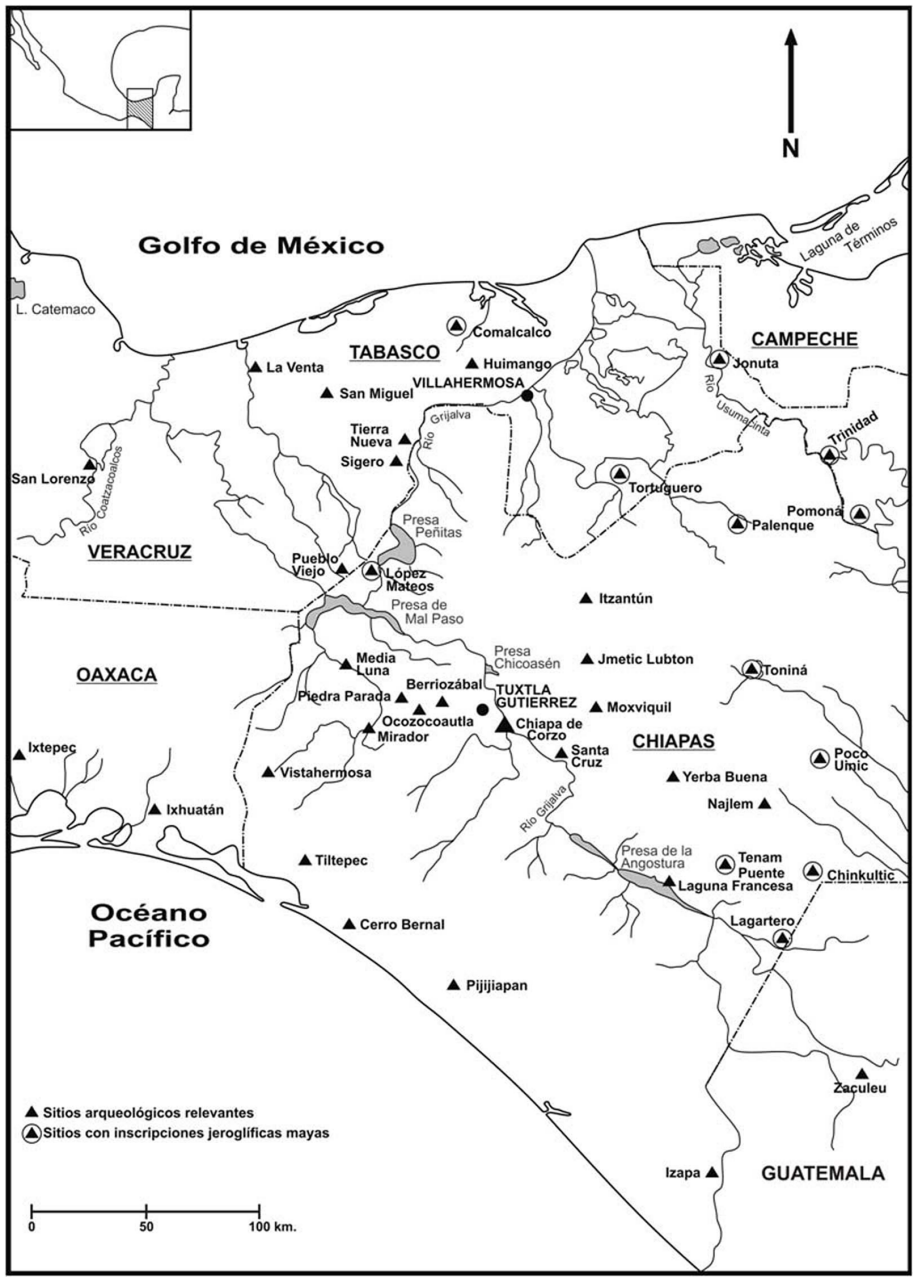
Map showing the location of Chiapa de Corzo in relation to the Preclassic world (Map made by Lynneth S. Lowe).

In the summer of 2012, while looking for the presence of cacao in a small set of pottery vessels from the site of Chiapa de Corzo, we identified chemical traces of *Capsicum* in five samples (Figure 1). Our initial objective for testing these pots was to confirm the notion that spouted jars, and associated vessel forms, were used in the production of cacao beverages as previously reported in the literature [17]–[22]. We also wanted to determine, if possible, whether cacao beverage were consumed plain or mixed with flavorings.

Research over the past decade into ancient Precolumbian use of cacao beverages has shown that spouted jars, generally utilized during the Middle and Late Preclassic (900 BCE-CE 300) periods across Mesoamerica, were utilized in the serving of this beverage [17]–[18], [20]–[24]. At present, there is limited information on the extent of types and forms of vessels used to prepare and/or serve cacao in different contexts (e.g., burials, caches, or from middens). There is also limited information on the methods and techniques used to prepare and serve cacao, whether these beverages were consumed by elite and non-elites alike, and whether the drinks were fermented. Regarding flavoring these cacao beverages, there is speculation that achiote, allspice, honey, maize, vanilla, zapote, various fruits, and/or chili pepper were added to provide different flavors and to make the bitter cacao beverages more palatable [17]–[18], [21], [25]–[29].

**Figure 2:**
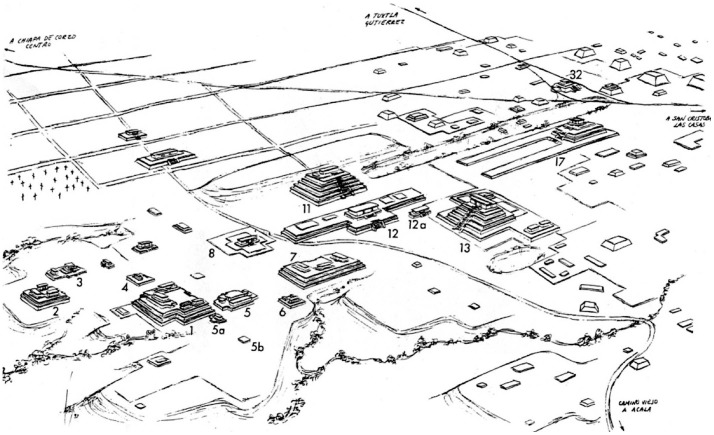
Site map of Chiapa de Corzo by CE 500 showing principle structures mentioned in text (Drawing by Ayax Moreno based on original map by Gareth W. Lowe).

Our study tested 13 intact pottery vessels obtained from stratified deposits excavated under the aegis of the Chiapa de Corzo Archaeological Project by the New World Archaeological Foundation (NWAF) between 1955 and 1963. The site is located near the modern city of Tuxtla Gutierrez, the capital of the State of Chiapas. Initial investigations by the New World Archaeological Foundation began at the site during the mid-1950s and have continued sporadically until today [30]–[34]. Chiapa de Corzo contains 81 structures, with numerous temples and palaces, mostly dating to the Late Preclassic (400 BCE-CE 300) period (Figure 2). The site was settled sometime approximately 1200 BCE by Mixe-Zoquean speakers who had strong ancestral and economic ties to Olmec people residing in the Gulf and Pacific Coastal regions of Mesoamerica. It reached its height between 700-200 BCE, when it was seen as a cultural intermediary between the Olmec and Maya civilizations.

We specifically chose vessels suspected to contain chemical traces of cacao as identified in previous studies at the sites of Colha located in northern Belize [20] and Puerto Escondido in Honduras [18], and vessels known to be associated with spouted vessels. Each of the vessels had been stored in a humidity-controlled environment since their excavation. Accession numbers were given to each pot from the site (Table 1). All of them were considered to be whole vessels prior to residue collection. They had all been washed prior to curation in the Museo Regional de Chiapas. Gratitude to the Consejo de Arqueologia in Chiapas for providing permission for our project to proceed and the exportation of the samples for analysis, as well as to Centro INAH Chiapas for providing the Chiapa de Corzo samples.

**Table 1:** Measurement data for the sampled pottery vessels from Chiapa de Corzo.

Sample #	*#* MRCH	# NWAF	Form	length/height	width	diameter
1	10-456167	2327	Tetrapod spout jar	15.5	16	10
2	10-457634	3397	Cylindrical vase	18	13.5	14
3	10-455436	1441	Jar with spout handle	15.5	13.5	9
4	10-250838	5254	Bowl	12	21	13
5	10-456304	2564	Tetrapod spout jar	8.5	9	4
6	10-250780	n/a	Polished white-slip urn	24.2	36.5	25.5
7	10-409571	3855	Sierra Red “florero”	33	19.5	18.5
8	10-250781	2643	Sierra Red “florero”	26.3	19.5	20
9	10-457648	n/a	Vase	24.2	14.5	16.8
10	10-456351	n/a	Tetrapod bowl	10.9	13.5	8.5
11	10-409637	284	Vase	14	12.6	13
12	10-457512	2643	Tetrapod spout jar	15.6	17	10.5
13	10-457518	3401	Vase	15.4	11.5	12.4

**Figure 3:**
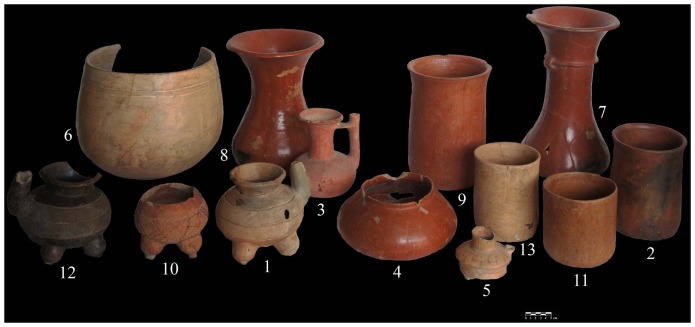
Image of the ceramic vessels from Chiapa de Corzo that were sampled for analysis (Photo by Roberto Lopez and Emiliano Gallaga Murrieta).

The sample included four spouted jars, four vases, three bowls, and two floreros (Figure 3, Table 1). All of the vessels are derived from Middle and Late Preclassic burial and cache offerings in Mounds 1, 5, 5A, 7, and 17. Five of the 13 vessels were excavated from the Mound 5 palace located in the center of the site. The 13 vessels were slipped either red, orange, cream, or black. In addition, they were also modified with either pre-slip incising and fluting or post-slip incising in a herringbone design. Some of the bowl forms had tetrapodal supports while the jars exhibited bridge-spouts.

### Residue Collection

No organic residues were visible on the interior surfaces of these vessels. Ancient Zoque pottery is low-fired, typically under 850°C, and therefore is ideal for absorbing and retaining organic compounds. While there were no visible organics adhering to the interior of these pottery fabrics, chemical extraction techniques were necessary for confirmation using a standardized technique [24]. The interior surface, taken primarily from the base and lower side walls, of each vessel was lightly scraped using a new piece of fine-grained sandpaper to remove any substances that may have permeated the vessel wall. Burr from each sample, ranging from 1–10 grams, was captured on a new sheet of multipurpose white paper and the material funneled into clean, previously unused collection vials and immediately sealed. New sheets of sand paper and multipurpose white paper were used for each sample collected. This method was rigorously upheld throughout the collection process to eliminate potential cross-contamination of sample materials. Following collection, sealed vials were sent to the Metabolomics Lab in the Department of Nutrition at the University of California, Davis for analysis.

## Laboratory Analysis

### Methods

Capsaicin, dehydrocapsaicin, 4-OH, 3-OMe-benzylamine, 3,4-Dihydroxybenzylamine and formic acid were purchased from Sigma-Aldrich Chemical Co. (St. Louis, MO). All solvents were mass spectrometry grade and all other chemicals used were of the highest grade available. Acquity UPLC HSS T3 1.7 µm (1×150 mm) column was purchased from Waters Corporation, Milford, MA. We confirm that we obtained permission from the Museo Regional de Chiapas to access the pottery vessels under analysis, and that they were loaned to us for one day while we conducted the residue study.

### Extraction of Chiapa de Corzo Pottery Samples

A total of 13 pottery samples were extracted using the following procedure. Ninety –200 mg of burr from each sample was vortexed with 1 ml methanol: chloroform mixture (1∶1) for 3 minutes then centrifuged. The resulting precipitate from each sample was removed and the supernatant was concentrated with Speed vac. To the residue 100 ul methanol: water (1∶1) was added, vortexted and filtered with 5 kD membrane filters. The Filtrates were transferred to vials for UPLC/MS-MS analysis.

### Ultra Performance Liquid Chromatography (UPLC)/MS-MS Analysis

**Figure 4:**
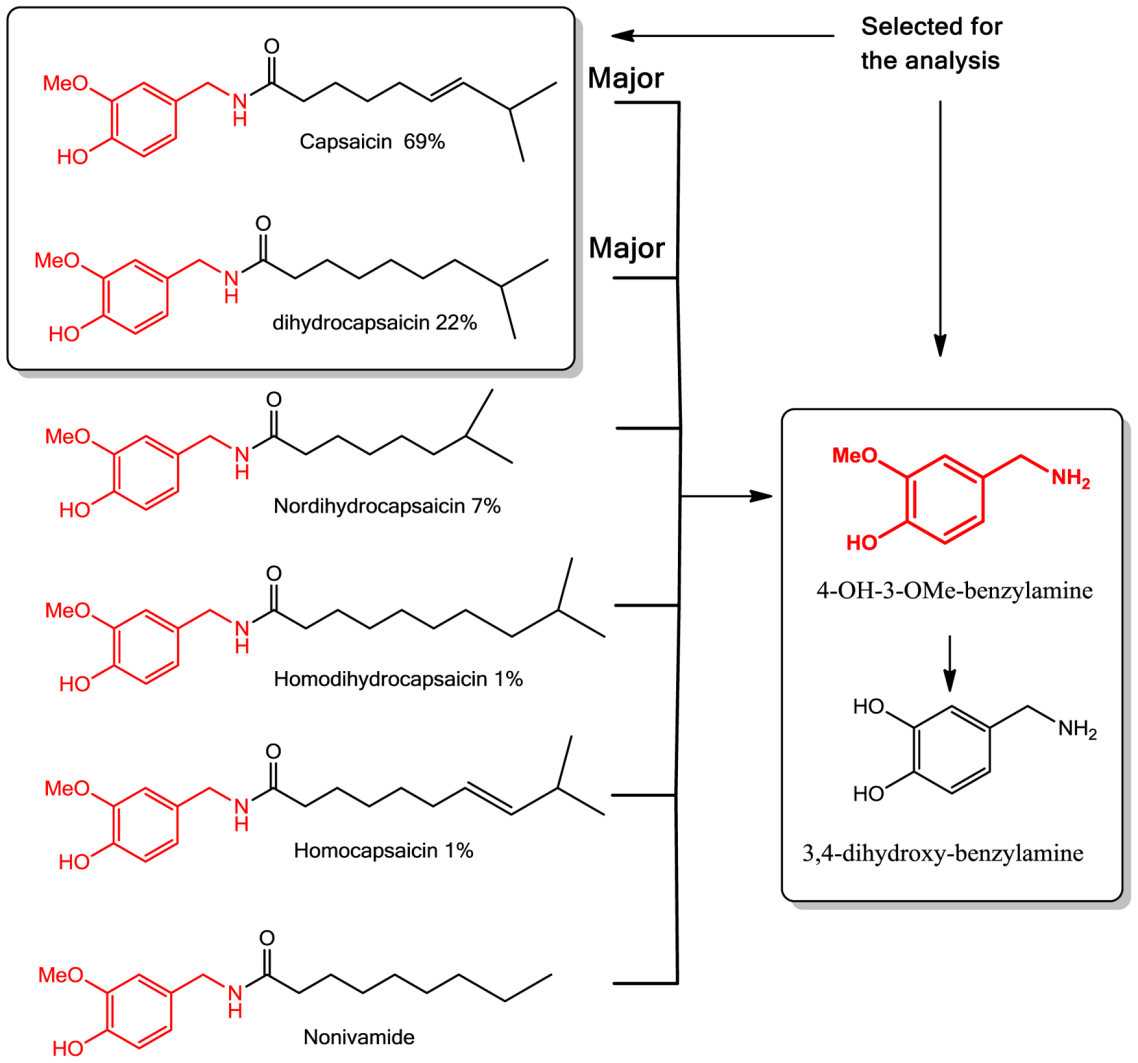
Major Capsaicinoids present in chili and proposed degradation pathway for biomarker analysis.

A Xevo-TQ triple quadruple mass spectrometer (Waters, Milford, MA, USA) was used to record MS and MS-MS spectra using Electro Spray Ionization (ESI) in positive ion (PI) mode, capillary voltage of 3.0 kV, an extractor cone voltage of 3 V, and a detector voltage of 500 V. Cone gas flow was set at 50 L/h and desolvation gas flow was maintained at 600 L/h. Source temperature and desolvation temperature were set to 150 and 350°C, respectively. The collision energy was varied from 6 to 13 to optimize four different daughter ions. The acquisition range was 20–350 D. Pure standards (Figure 4) (Capsaicin, dehydrocapsaicin, 4-OH, 3-OMe-benzylamine and 3, 4-Dihydroxybenzylamine) were introduced to the source at a flow rate of 10 µl/min by using methanol:water (1∶1) and 0.1% formic acid mixture as the carrier solution to develop multiple reaction monitoring (MRM) method for UPLC/MS-MS operation (Table 2).

**Table 2:** MRM method parameters used in chilli analysis.

Analyte	mode	Parentm/z	Daughterm/z	Cone	Coll
Capsaicin	PI	306.3	137.1	20	13
Dihydrocapsaicin	PI	308.3	137.0	19	11
4-OH,3-OMe-benzylamine	PI	154.1	137.1	8	4
3,4-Dihydroxybenzylamine	PI	140.1	123.1	10	6

UPLC/MS-MS analyses of all the samples were carried out with a Waters Acquity UPLC system connected with Xevo-TQ triple quadruple mass spectrometer. Analytical separations on the UPLC system were conducted using a Acquity UPLC HSS T3 1.7 µm column (1×150 mm) at a flow rate of 0.15 ml/min. The gradient started with 100% A (0.1% formic acid in H_2_O) and 0% B (0.1% formic acid in CH_3_CN), changed to 50% A over 3 min, followed by a 4-min linear gradient to 10% A, resulting in a total separation time of 7 min. The elutions from the UPLC column were introduced to the mass spectrometer and resulting data was analyzed and processed using MassLynx 4.2 software. Pure standard mixture was used to optimize the UPLC conditions prior to analysis. After LCMS analysis the remaining extract of the samples was stored at −80°C in the Metabolomics Lab in the Department of Nutrition at UC-Davis for further evaluation.

## Results

**Figure 5:**
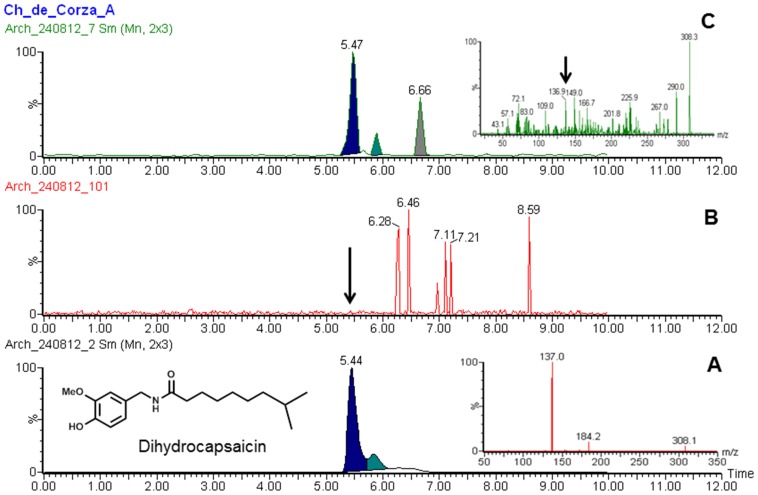
UPLC/MS-MS chromatograms illustrating (a) Standard dihydrocapsaicin (b) Blank (c) Representative Corza sample confirming the presence of dihydrocapsaicin. Insets: MS/MS spectra of standard dihydrocapsaicin (A) and from sample extract (B). Samples were extracted and analyzed as described in methods.

**Figure 6:**
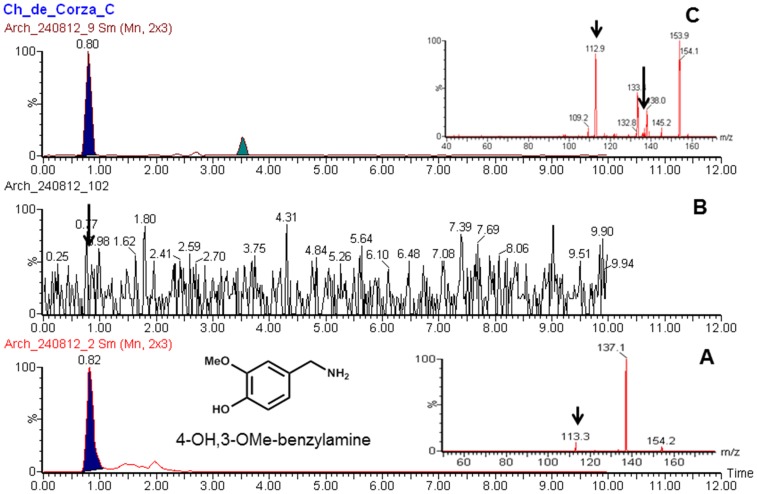
UPLC/MS-MS chromatograms illustrating (a) Standard 4-OH-3OMe-benzylamine (b) Blank (c) Representative Corza sample confirming the presence of 4-OH-3OMe-benzylamine. Insets: MS/MS spectra of standard 4-OH-3OMe-benzylamine (A) and from sample extract (B). Samples were extracted and analyzed as described in methods.

**Figure 7:**
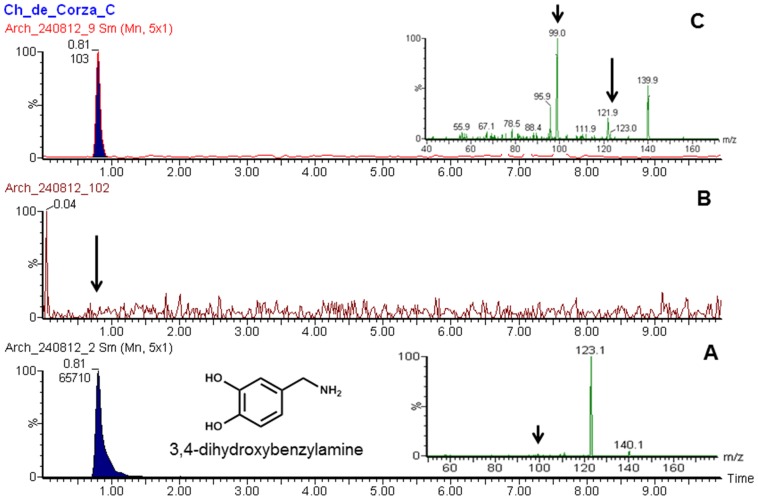
UPLC/MS-MS chromatograms illustrating (a) Standard 3,4-dihydrobenzylamine (b) Blank (c) Representative Corza sample confirming the presence of 3,4-dihydrobenzylamine. Insets: MS/MS spectra of standard 3,4-dihydrobenzylamine (A) and from sample extract (B). Samples were extracted and analyzed as described in methods.

The UPLC/MS-MS analyses of Chiapa de Corzo samples clearly shows a presence of peak at 5.47 min (Figure 5c) that matches well with the standard dihydrocapsaicin (Figure 5a). Furthermore, the samples shows a presence of peak at 0.80 min (Figure 6c) that matches well with the standard 4-OH, 3-OMe-benzylamine (Figure 6a). Similarly, peak at.81 min (Figure 7c) matched well with the standard 3, 4-Dihydroxybenzylamine (Figure 7a). Moreover, there were no corresponding peaks seen in blank (Figure 5b, 6b and 7b). Additionally, no peaks were seen in Corzo samples that corresponded with the standard capsaicin peak (data not shown). Table 3 shows all the dehydrocapsaicin, 4-OH, 3-OMe-benzylamine and 3,4-Dihydroxybenzylamine positive Corzo samples (2, 3, and 7), which confirms presence of chili. The traces amount of 4-OH,3-OMe-benzylamine was found in 1, 6, 8, 9, AND 12 samples, whereas trace amount of 3,4-Dihydroxybenzylamine was present in 6 and 7. The rest of the Chiapa de Corzo samples did not show presence of detectable peaks at 5.44, 0.82 and 0.81 min. reaction monitoring (MRM) method for UPLC/MS-MS operation (Table 2).

**Table 3:** UPLC-MS/MS analysis of pottery samples.

Sample#	Capsaicin	Dihydrocapsaicin	4-OH,3-OMe-benzylamine	3,4-Dihydroxybenzylamine	Chili status
**1**		+	t		Positive
**2**			+		Positive
**3**			+	+	Positive
**4**					
**5**					
**6**			t	t	
**7**			+	t	Positive
**8**			t		
**9**			t		
**10**					
**11**			t	+	Positive
**12**			t		
**13**		t			

+ = identified, t = traces.

### Chili Pepper-positive Pottery

**Figure 8:**
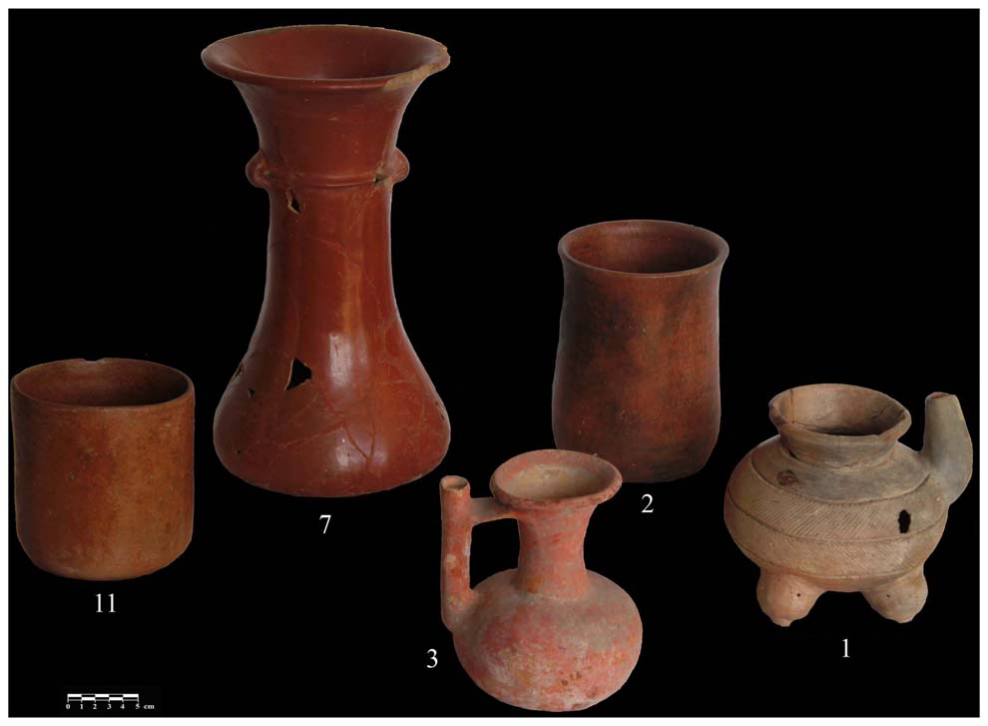
Image of the five vessels that tested positive for *Capsicum* from Chiapa de Corzo. Photos by Roberto Lopez and Emiliano Gallaga Murrieta.

The results of these analyses provide conclusive evidence for the presence of *Capsicum* spp. in five (Vessel 1,2,3,7, and 11) of the 13 samples (Figure 8). Sample 2 represents the earliest positive chemical signature and confirms early chili pepper consumption at the regional ceremonial center of Chiapa de Corzo by 400 BCE (Francesa Phase). Vessels 1 and 3 are dated slightly later to ca. 200 BCE (Guanacaste Phase) while Vessels 7 and 11 are derived from contexts dated from CE 100–300 (Horcones Phase). Four of the five positive vessels (1, 2, 3, and 11) were found in Mound 5 in different contexts but all of them are associated with high status individuals. For example, Sample 2 was found in a massive burial offering and Sample 1 from a rich offering inside Room 8. Mound 5 is a palace structure located on the southern portion of the site. Mound 5is 32 meters long by 30 meters wide and has a maximum height of two meters. There are several entrances on the structure, delimited by pillars, which open onto courtyards where archaeologists think that the Chiapa lords lived or at least held audiences. At least five sumptuous tombs have been found in this palace structure, one of which (Burial 149) contained one of the earliest chili-positive pottery vessels (Sample 2) [35]–[38].

Vessel 1 is a short, polished brown tetrapod free-spout jar with incised herringbone decoration located around the body. Vessel 2 is a tall, narrow, orange-slipped cylindrical vase with no decoration on its exterior surface. This vessel, with the earliest chemical signature of *Capsicum*, was found in a grave of an adult male who was buried with 14 other ceramic pots, jade earplugs, and a bivalve sea shell pectoral necklace. Vessel 3 is a tall, orange-slipped bridge-spout jar with a globular body. Vessel 11 is a short, wide, orange-slipped vase with no exterior decoration. The other positive sample, Vessel 7, was found in Tomb 7 in Mound 1, which is contemporaneous in date with Mound 5. Mound 1 is located at the south end of Chiapa de Corzo. It is identified as a temple complex used for religious activities. In this structure, at least five elite tombs were uncovered. The lords interred were richly adorned with sumptuous offerings, including several imported vessels from Guatemala and Oaxaca, as well as from Usulután, El Salvador [30], [35]–[38]. Vessel 7 is a tall, red-slipped vase-like pot that is often referred to as a “florero” among Zoquean archaeologists.

## Discussion and Conclusions

The present study initially was conducted to search for chemical traces of cacao (*Theobroma cacao*) in a variety of vessel forms (e.g., bowls, jars, and vases), and most notably spouted jars, which were recovered from a variety of contexts (e.g., burials, caches) at Chiapa de Corzo. We also were interested in identifying flavorings and/or seasonings that could have been added to the cacao beverages. While we sought to recover chemical traces of cacao, none were found. We identified, however, traces of *Capsicum* in 38% of the sampled pottery. The results of the chemical extractions provide definitive proof that Middle and Late Preclassic vessels from the site contained *Capsicum*. The information obtained from the Chiapa de Corzo vessels extends the date of chili pepper use by the ancient Mesoamerican peoples by several hundred years into the Middle Preclassic period to about 400 BCE.

While our scientific study has pushed back the antiquity of Mesoamerican chili pepper presence, we are most interested in how the pepper may have been used either from a culinary, pharmaceutical, or ritual perspective during this early time period. Finding positive *Capsicum* evidence in five samples of different pottery types and shapes – and where *Capsicum* was the only residue identified – a number of questions may be raised. It is important to mention that the analyses conducted on these samples were repeated to verify the presence of *Capsicum* in the positive samples.

Was the chili ground up to produce a paste or a salsa and subsequently used as a seasoning in foods that were offered to the Zoquean gods or chiefs? Or, were the peppers left whole in the pots? We assume that the presence of chili is in the form of a sauce or paste, and not whole given that no seeds or other macrofossils were identified in the interiors of the vessels. If the peppers were served in paste form, then testing grinding implement (e.g., manos and metates) residues would be the next fruitful step in understanding the culinary practice involved. A starch grain analysis of such food preparation implements would complement our chemical extraction technique conducted on the pottery vessels. All of the vessels that tested positive for *Capsicum* were serving vessels and it becomes important to identify which pots may have been used in mixing the peppers into a paste form. At present, we do not have access to a full range of vessels associated with making such a seasoning. However, to date, there is not a single “molcajete’ found in the Chiapa de Corzo ceramic collection for the Preclassic period, like those found in Central Mexico for salsa making in later periods. This vessel form is found at Chiapa de Corzo but for the Postclassic period [34].

Further, were chili peppers consumed by elites and non-elites alike? All the chili-positive pottery vessels analyzed were discovered in different elite contexts from a long span of time from 400 BCE through CE 300, so was there restricted use of this pungent fruit 2,400 years ago? To address this question we would need to expand the dataset to include vessels derived from lower status or commoner contexts such as middens and burials.

Why would there be evidence of chili peppers in a spouted jar? It is commonly assumed that spouted jars were used for pouring a liquid into another container. Perhaps the peppers were not made into a sauce but a spicy beverage or alternatively a chili sauce that was stored in the spouted jars and subsequently poured as a dining condiment. Given the pungency and “heat” associated with chili preparations, it would be logical to suggest that specific jars would be used to store and serve the products – jars whose only function was to store and serve chili-related products, at least for Samples 1 and 3. It is important to consider the possibility that all or at least some of the pots deposited at a specific event (tomb or offering) would be part of the community-wide feast and that afterwards the empty pots were deposited as part of the ritual deposition [24], [39]. If so, then the drained pots would leave minimal residues for archaeologists to examine.

The sampled vessels were chosen by form rather than context, making the assumption that those particular types would be more likely to get a positive result for cacao (*Theobroma cacao*). The unexpected results provide a different scenario. From five different elites contexts (three burials: Tomb 7, 12, and 149, and two offerings) and each of them containing several vessels, at least one of the vessels chosen had chili residue on it. It would be interesting to sample the entire vessel assemblage from one or more of these contexts to see if other vessel types and forms had chili in them, or if other substance(s) were identified such as cacao. Information from hieroglyphic texts, both on vessels and murals, will help to shed light on the types of beverages prepared and consumed as well as which ingredients were used to flavor them. There is strong evidence for this in the Maya area where epigraphers have deciphered texts showing different kinds of cacao-based beverages and gruels [40]–[43], for example, but, to date, few identify chili peppers as an additive or flavoring. It is hoped that we can remedy this lack of information, from a biomolecular perspective, in the future when we conduct an indepth study of the remaining pottery vessels from Chiapa de Corzo.

Is it possible that the chili substance inside these vessels was used for medicinal, ritual, or magical purposes rather than culinary? Recent studies show that *Capsicum* species were included in a number of herbal remedies for microbial origin ailments by the Maya [44]. Once again we have the problem that only *Capsicum* was identified, but still possible that the chili residue on the pot was placed to be used for medicinal purposes by its owner in the other world.

Another explanation for this unexpected result is provided by Alfredo Lopez Austin. He suggested that sometimes the interior of the vessels were covered with a mixture of chili and ash as a measure to prevent/repeal insects to eat whatever is inside (Alfredo Lopez Austin, personal communication, 2013). So, in this case the chili could be just a part of the process to preserve something else that was not preserved or taken away at some point in time.

The fortuitous finding of *Capsicum* species in these pots provides the earliest evidence of chili consumption in well-dated Mesoamerican archaeological contexts. We do not know exactly what the Chiapa de Corzo people were doing with it, but are clear that its use is important to be present on at least five different elite contexts and to be part of a ritual paraphernalia from at least 400 BCE to AD 300. Clearly, more research would be necessary to know what exactly *Capsicum* species are been used for, if it is a local species or is part of the well-known Mixe/Zoque trade network at Chiapa de Corzo. These questions, among others, would be promising but spicy areas of future research.
